# Walking away from depression: the mediating role of walking activity in depression impacting blood glucose levels of people with diabetes or prediabetes

**DOI:** 10.3389/fendo.2024.1446405

**Published:** 2024-08-27

**Authors:** Yifat Fundoiano-Hershcovitz, Inbar Breuer Asher, Halit Kantor, Sandy Rahmon, Marilyn D. Ritholz, David L. Horwitz, Omar Manejwala, Pavel Goldstein

**Affiliations:** ^1^ Clinical Department, Dario Health, Caesarea, Israel; ^2^ School of Public Health, University of Haifa, Haifa, Israel; ^3^ Joslin Diabetes Center, Harvard Medical School, Boston, MA, United States; ^4^ Clinical Department, DLH Biomedical Consulting, Las Vegas, NV, United States; ^5^ Commercial-Medical Department, Dario Health, Caesarea, Israel

**Keywords:** blood glucose, physical activity, steps counting, depression, mediation effect, digital health, diabetes

## Abstract

**Introduction:**

Depression can exacerbate diabetes by impairing self-care behaviors and increasing the risk of complication; however, the underlying mechanism is still unclear. Given the suggested associations between walking activity, depression status, and blood glucose levels this study explores the intricate relationship between depression and blood glucose (BG) control, with a focus on walking activity as a behavioral mediator. The purpose of this study is to examine walking activity’s mediating role in depression’s impact on BG levels, investigating and validating the non-linear association between BG levels and walking activity. This retrospective real-world study demonstrates the potential of regular walking activity as a simple and accessible intervention to mitigate the negative effects of depression on BG levels in T2D and prediabetes.

**Methods:**

A cohort of 989 users with T2D and prediabetes, who regularly tracked their steps levels and BG levels for 12 months using the Dario digital health platform was evaluated. The mediating role of the monthly average number of steps on the relationship between the self-reported depression status and lagged monthly average BG was assessed. Additionally, the association between monthly walking activity and monthly average BG was tested using a piecewise linear mixed effects model.

**Results:**

Users with self-reported depression demonstrated increased BG levels compared to users without depression (B=8.00, P=.01). The association between depression and monthly average number of steps was significant (B=-.27, P<.005) and monthly average number of steps significantly predicted the following months’ average BG (B=-.81, P=.001), adjusting for depression. The monthly average number of steps significantly mediated the effect of self-reported depression on the following month’s average BG (M=.22, P<.005). Further sensitivity analysis demonstrated model robustness over various periods. Finally, non-linear dynamics of walking activity over time was validated using unseen data showing a decrease in monthly average BG for users with over an average of 400 steps per day (B=-1.87, P<.01).

**Discussion:**

This study shows how regular walking may reduce the negative impact of depression on BG levels in people with T2D. Our findings advocate for the integration of walking activity into treatment protocols as a cost-effective, accessible intervention strategy to improve glycemic management and depressive symptoms in this population.

## 1 Introduction

The co-occurrence of depression in individuals with T2D is a well-documented phenomenon, with about 18%–25% of patients with T2D experience significant depressive symptoms (1, 2). This interrelation poses a significant challenge to the management of diabetes, as depression can exacerbate diabetes outcomes by undermining self-care behaviors and increasing the risk of diabetes-related complications (2–4). The intricate relationship between mental health and metabolic regulation in diabetes highlights the critical need for comprehensive strategies that address both psychological and physiological components of diabetes management through mindset shifts and behavior modifications. The capacity for physical activity can be behaviorally regulated by adopting a positive mindset and modifying daily habits, fostering a proactive approach to incorporating exercise into daily routines (5). Physical activity is a cornerstone in the management of type 2 diabetes (T2D) and prediabetes, recognized for its capacity to improve glycemic control, facilitate weight management, and enhance overall well-being (6–10). Exercise is beneficial to the general population and in people with prediabetes as it lowers the risk of developing T2D (10, 11). Among various forms of physical activity, walking is one of the easiest applicable forms of exercise due to its accessibility, low cost, and minimal requirement and may represent, for many, a first step towards lifestyle change (12). Focusing on walking aligns with the study’s aim to explore how this accessible, low-cost and easily integrated physical activity can mediate the relationship between depression and blood glucose levels among users with T2D and prediabetes. It underscores the potential of walking as a feasible and effective intervention strategy in promoting both physical and mental health in this population. Walking has been associated with significant health benefits, including improved blood glucose levels and reduced cardiovascular risk factors (13). The American Diabetes Association recommends that people with diabetes start exercising slowly by gradually progressing to a brisk walk, and monitoring both the duration and number of steps taken. Regular walking reduces A1C, triglycerides, blood pressure, and insulin resistance and intensive walking promotes rapid enhancement of skeletal muscle oxidative capacity, insulin sensitivity, and glycemic management (fasting blood glucose) (14, 15). Even a moderate level of walking can show long-term effects on glycemic improvement, as measured by HbA1c (16, 17). There is limited knowledge on the relationship between number of steps walked and diabetes risk or clinical outcomes, as well as on how the number of daily steps walked impacts blood glucose levels over time (18, 19). Most of the existing data were derived from self-report or survey tools or RCTs that, suffered from a number of limitations such as generalizability (20, 21). More longitudinal research on the association of daily step counts with clinical outcomes using real-life data are required (18, 20, 22).

Furthermore, walking activity has been proposed as a therapeutic strategy not only for its physical health benefits but also for its potential psychological benefits, including alleviating symptoms of depression (23–25). Evidence for the efficacy of walking as an intervention for depressive symptoms has been found in several meta-analyses (23, 26, 27). A variety of mechanisms have been suggested as to why physical activity and might reduce depressive symptoms (23) such as biochemical, physiological, psychological and psychosocial (28). Nevertheless, previous studies have not been able to identify the optimal characteristics for the design and delivery of a walking intervention that can benefit depressive symptoms (29).

Studies have shown that engagement with digital health apps and using activity trackers for walking activity have positive effects on physical activity, physiological metrics and psychosocial outcomes (30). Previous evidence demonstrated that using smartphone apps for tracking and promoting physical activity and using pedometers can enhance engagement and increase efficacy of interventions. However, limited knowledge is available about the effect of long-term walking on diabetes outcomes in real-life settings (31, 32).

Specifically, previous research highlights the effect of depression on glycemia (3, 30, 31). However, walking activity may potentially modulate this effect in T2D based on the biopsychosocial model, which posits that biological, psychological, and social factors have significant, interdependent impacts on health (33). Depression, a common comorbidity in T2D, is known to negatively affect self-management behaviors, including adherence to dietary recommendations, medication regimens, and physical activity guidelines. This reduction in self-care activities can lead to poor glycemic management, creating a vicious cycle of worsening depressive symptoms and diabetes management (3, 34, 35).

In this complex interplay, walking activity emerges as a promising behavioral intervention that might bridge the gap between mental and physical health. The act of walking could potentially counteract the negative effects of depression on diabetes management by improving mood, enhancing self-efficacy, and directly contributing to better blood glucose levels through increased physical activity. These effects are likely to be mediated by a range of biological and psychological mechanisms, including the release of endorphins, improvements in insulin sensitivity, and the establishment of a routine that fosters a sense of accomplishment and control over one’s health (36–39).

This study aims to assess the relationship between depressive symptoms and blood glucose levels in individuals with T2D over time, with a specific focus on walking activity as a potential behavioral mediator. In addition, the study aims to investigate the association between monthly aggregated blood glucose (BG) measurements and walking activity (number of steps), by testing potential non-linearity.

## 2 Methods

### 2.1 Platform

**Figure 1:**
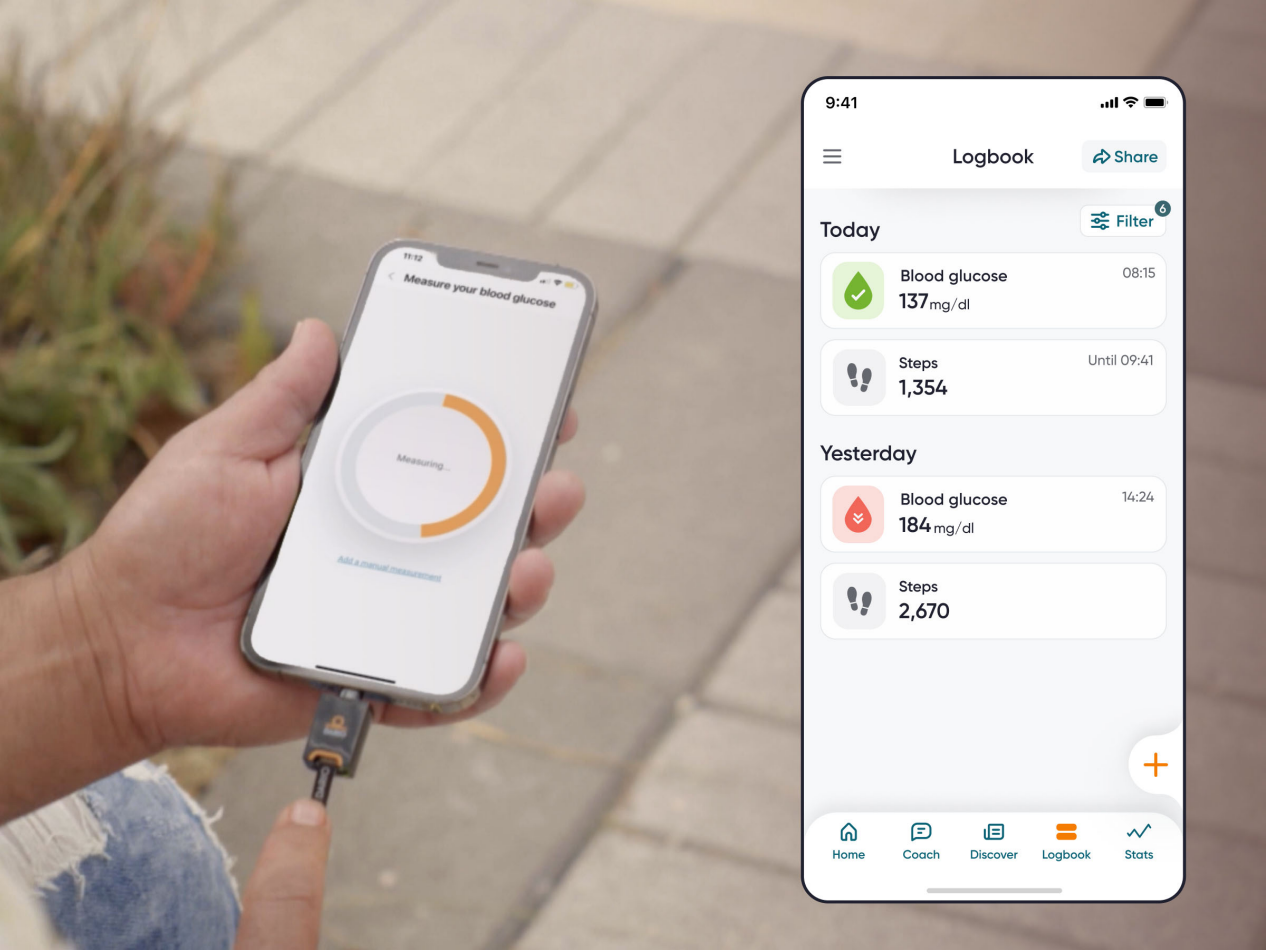
A BG measurement taken on the Dario mobile app platform via the glucose meter and a logbook screen presenting BG measurements and daily number of steps.

This study used the Dario digital therapeutics platform (Dario Health) for chronic conditions to support self-management of diabetes. The glucose meter consists of a small, pocket-size holder for strips, a lancet, and the meter. The BG meter is removed from the holder and plugged directly into a smart mobile device, effectively converting the smart mobile device into a display screen for the meter (**Figure 1**). Data is uploaded to the cloud for backup and further analysis. Step counts were recorded within the app through either the integrated pedometer of a smart mobile device or by interfacing with Apple Health.

### 2.2 Measures

The monthly average blood glucose was the first measure. The monthly average blood glucose was defined as the mean of all blood glucose measurements taken over a 30-day interval for each user. It was used as the core outcome metric to reflect the monthly aggregated interval changes over 12 months. As part of the app’s intake questions, we also gathered demographic information, including gender, age, weight, and height. Additionally, we collected self-reported medical data through the digital health platform. This data included diabetes type and other comorbid conditions such as depression, by having users select the relevant condition from a comprehensive dropdown list of comorbidities. By utilizing a user-friendly dropdown list we ensured consistent data collection, enabling robust analysis of the interplay between diabetes, comorbid conditions, and overall health outcomes.

### 2.3 Study population

The inclusion criteria required Dario platform users to have consistently measured their blood glucose every week for a full year between 2019 to 2023 and to have reported type 2 or prediabetes in their diabetes type within the app. Additionally, users were required to have an average daily step count exceeding 10 steps. No exclusion criteria were applied. Subpopulation included users who reported Depression in the app. This study is based on an analysis of an existing database already collected in the digital platform. All data were anonymized before extraction for this study. Ethical and Independent Review Services, a professional review board, issued the institutional review board exemption for this study (18032-06#) (40).

### 2.4 Study design

We applied a retrospective longitudinal cohort study design utilizing a database containing previously recorded follow-up data from the Dario Health users.

### 2.5 Statistical analysis

In this study, we used a mediation model based on a mixed model framework to infer quasi-causal relationships between depression status and BG levels through walking activity. To ensure the robustness of our findings, we carefully controlled for potential confounding variables and considered the temporal relationships among the variables.

Firstly, we identified and included relevant confounding variables in the mediation model, such as age, gender, diabetes type, diabetes duration, insulin treatment, and weight. By including these confounders, we aimed to isolate the specific pathways through which depression status may affect BG levels.

Secondly, our mediation analysis uses longitudinal data to account for temporal relationships. By analyzing data collected at multiple time points, we were able to establish the temporal sequence of events, which is crucial for causal inference using 1 month lag in the measures of walking activity and BG levels. This approach helps to ensure that the mediator precedes the outcome, thereby supporting the causal interpretation of the mediation effects.

Furthermore, we used sensitivity analyses to assess the robustness of our mediation findings, exploring the mediation effect across various time points. The results of these sensitivity analyses suggested that our mediation model is stable over time, further strengthening our causal inferences.

Overall, by controlling for relevant confounders, considering temporal relationships, and conducting sensitivity analyses, we aimed to ensure the validity and reliability of our mediation analysis and the quasi-causal inferences drawn from our study.

Here, we applied the following models: (a) examining the effect of depression status on the monthly average number of steps; and (b) examining the association between monthly average number of steps a day and 1-month lagged monthly average blood glucose, conditioned on the depression status. The mediation effect was defined as the a*b and statistical inferences were made based on the approach described above. The mediation effect was tested using a quasi-Bayesian–Monte Carlo method with 5,000 simulations. A quasi-Bayesian–Monte Carlo method for testing the mediation effect is particularly suited for longitudinal data due to its ability to incorporate prior information and handle complex data structures. This method involves generating random samples from the posterior distribution of the mediation effect through Monte Carlo simulations, allowing for accurate estimation of the mediation effects while accounting for uncertainties and potential biases.

The quasi-Bayesian–Monte Carlo method offers several advantages:

1. It effectively deals with the longitudinal nature of the data, allowing for time-varying exposures and mediators. For example, Mittinty and Vansteelandt (2020) demonstrated the robustness of Bayesian methods to unmeasured confounding in longitudinal mediation analysis​ (41).
2. The Bayesian approach allows us to incorporate prior knowledge into the analysis, improving the robustness and interpretability of the estimates. Studies like those by Gao and Albert (2019) have shown the advantages of Bayesian methods in handling complex data structures for longitudinal mediation models​ (42)​.
3. The Monte Carlo simulations provide accurate approximations of the mediation effects and their uncertainties, even with complex models and data distributions. Vansteelandt et al. (2019) successfully applied Bayesian dynamic mediation analysis to account for time-varying exposures and mediators, supporting the method’s suitability for longitudinal studies (43).

Furthermore, a sensitivity analysis to the mediation effect was performed to evaluate stability in time. We used moderated mediation models assuming fluctuations of the mediation effect over time by adding interaction effects between time and depression. We first tested whether the association between depression and monthly average number of steps varied across time. Then we tested whether the previous month’s average number of steps is associated with the monthly average BG across time, conditioned on the depression effect modulated by time. Finally, we tested the mediation effects at specific time points: months 2, 5, 10 and 12.

A classic linear longitudinal model assumes a single-slope growth pattern for changes in an outcome variable across time. In contrast, piecewise‐based mixed‐effects models allow for the modeling of variable change trajectories across time, accommodating potential non-linear relationships between walking activity and blood glucose levels. Here, a piecewise linear mixed effects model was applied to model the trajectories of monthly average BG levels and monthly average number of steps a day over a year among users with and without depression.

The monthly average number of steps was distributed right skewed; therefore, a log transformation was applied to fit the models’ assumptions. In the field of physical activity, outcome variables are often count variables (a non-negative integer) and therefore are commonly positively skewed (44). When fitting a linear mixed-effects model, assuming a normal distribution for this type of data can be problematic (45), as it does not match the characteristics of the model and may affect the interpretation and conclusions (44). Therefore, it is common to analyze this type of data on a transformed scale that yields an approximately normal distribution, and a log transformation is reported as the most popular due to its ease of use and interpretability (44, 45).

The threshold of 400 steps per day was selected based on a detailed exploratory data analysis, which included visualizing the relationship between average monthly steps and BG levels. The visualization revealed a noticeable inflection point at approximately 400 steps per day, beyond which the association between steps and BG levels appeared to change. This threshold was chosen to account for this observed non-linearity and to improve the model’s ability to detect meaningful patterns in the data. Following a visualization of the association between monthly average steps and BG, the average steps were divided into 2 segments (<400 steps a day/>400 steps a day), assuming a change in BG levels at >400 steps a day. Overfitting is a condition that occurs when a model that shows a good fit for a specific data set then cannot be generalized to a new data. Therefore, the data set was randomly partitioned (by users) into 70% train set, which will actually be used to adjust the parameters of the models, and 30% test set which will be used to measure the predictive validity of the chosen model during the training.

## 3 Results

### 3.1 Users

In total, a group of 989 users were included in this analysis. The study cohort comprised 55% (546/989) of men, the average age was 62.5 (SD ±12.7) and average BMI was 32.5 (SD ±6.9). The average diabetes duration was 9.4 years (SD ±8.9). A total of 14% (140/989) of the users reported having depression, 82% (808/989) have T2D and 18% (181/989) have prediabetes. Of the users, 78% (771/989) reported not using insulin, 22% (216/989) reported using an insulin pen and only 2 users reported using an insulin pump.

### 3.2 Effect of depression on blood glucose levels and walking activity over time

**Figure 2:**
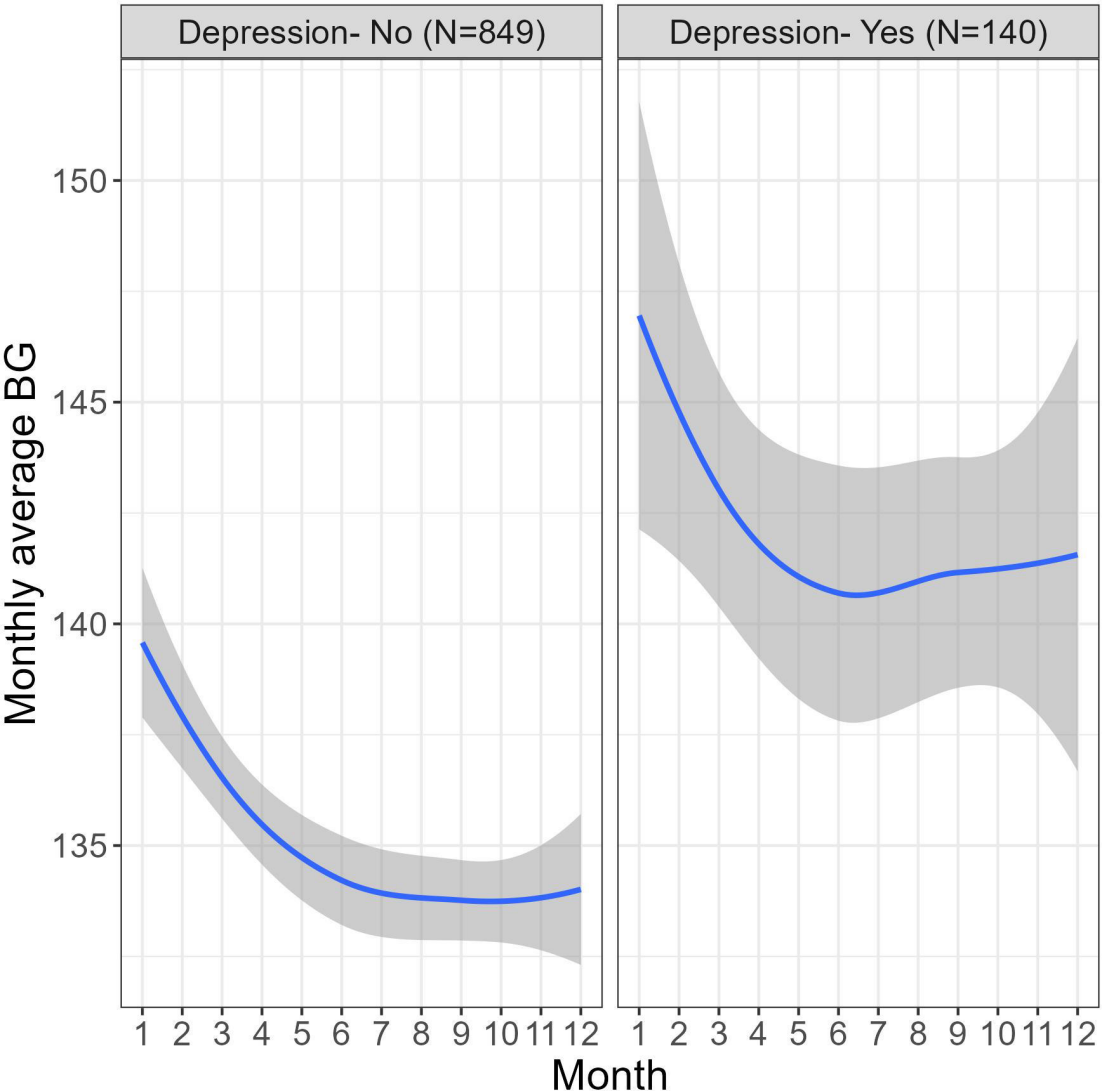
Differences in monthly average BG (mg/dL) fluctuations across 12 months of platform usage in users with and without depression.

**Figure 2** demonstrates a similar time-associated behavior in the monthly average BG among users with and without depression yet the BG levels in the depression group are higher across the 12-month period.

Piecewise mixed model analysis was used to test the differences in the trajectories of the monthly average BG during two periods- 1-4 months and 4-12 months. A significant decrease in the BG levels was found during the first 4 months of platform usage (B=-1.83, 95% CI [-1.71, -1.06], P<.001), in months 4-12 there was no significant change in the BG levels (B=.00, 95% CI [-0.15, 0.15], P=.99). Fifty-nine percent of users showed significant overall decrease in their BG levels over the 12 months of platform usage (t(989) = –8.03, P<.001). Moreover, the ratio of users with monthly average BG below 117mg/dL (equivalent to A1c 5.7, indicating a cut-off of normal blood glucose levels (46)) increased by 33% over a year.

Also, a statistically significant association was found between the depression status and BG levels (B=8.00, 95% CI [1.93, 14.06], P=.01), meaning that users with depression have higher avg BG levels than users without depression over a 12-month period. The follow-up time did not moderate the effect, meaning that the trajectories of the monthly average BG in both periods were similar in both users with and without depression (months 1-4: B=-.39, 95% CI [-1.25, 0.46], P=.37 and months 4-12: B=.25, 95% CI [-0.16, 0.66], P=.23).

Another piecewise mixed model analysis was conducted to test the differences in the trajectories of the monthly average number of steps (following a natural log transformation) during two periods- 1-7 months and 7-12 months, in users with and without depression. Also, a statistically significant association was found between the depression status and monthly average number of steps (B=-.57, 95% CI [-0.78, -0.36], P<.001), meaning that users with depression have significantly lower average number of steps than users without depression over a 12-month period. A significant interaction effect was found between depression status and the first trajectory, 1-7 months (B=.03, 95% CI [0.01, 0.04], P<.001), the interaction between depression and the second trajectory, 7-12 months was insignificant (B=-.02, 95% CI [-0.04, 0.], P=.05). No significant changes in the monthly average number of steps were found during the first 7 months of platform usage (B=.00, 95% CI [-0.00, -0.01], P=.07), in months 7-12 a significant decrease was found (B=-.01, 95% CI [-0.02, -0.01], P=.001).

### 3.3 Mediating role of walking activity

The association between depression and monthly average number of steps was significant regarding the differences between the two groups (B=-.27, 95% CI [-0.44, -0.10], P<.005). Users with type 2 diabetes compared to users with prediabetes (B=-.17, 95% CI [-0.33, -0.01], P<.05) and those with insulin pens compared to those who do not use insulin (B=-.27, 95% CI [-0.42, -0.13], P<.001) had significantly lower monthly average number of steps, while users with insulin pumps did not have significant differences in monthly average number of steps (B=-.08, 95% CI [-1.35, 1.18], P=.90) compared to non-insulin users. In addition, a negative association was found between age (B=-.02, 95% CI [-0.03, -0.02], P<.001) diabetes duration (B=-.27, 95% CI [-0.42, -0.13], P<.001) and weight (B=-.01, 95% CI [-0.01, -0.01], P<.001) with the monthly average number of steps. It was also found that men had more monthly average number of steps compared to women (B=.61, 95% CI [0.49, 0.74], P<.001).

Adjusting for the previous months’ average number of steps is negatively associated with the monthly average BG (B=-.82, 95% CI [-1.31, -0.32], P=.001), conditioned on the effect of the depression (B=2.73, 95% CI [-2.33, 7.79], P=.29) which was not significant. Users with type 2 diabetes compared to users with prediabetes (B=14.3, 95% CI [9.52, 19.04], P<.001) and users with insulin pens compared to non-insulin users (B=16.55, 95% CI [12.22, 20.88], P<.001) had significantly increased monthly average BG levels, while users with insulin pumps compared to non-insulin users did not have significant differences in monthly average BG (B=20.77, 95% CI [-17.06, 58.61], P=.28). In addition, it was found that diabetes duration (B=0.03, 95% CI [0.01, 0.05], P<.005) and weight (B=0.17, 95% CI [0.10, 0.25], P<.001) were associated with increased monthly average BG. Age and gender were not significantly related to monthly average BG (B=-.04, 95% CI [-0.20, 0.11], P=.59 and B=-3.26, 95% CI [-6.93, 0.40], P=.08, respectively).

**Figure 3:**
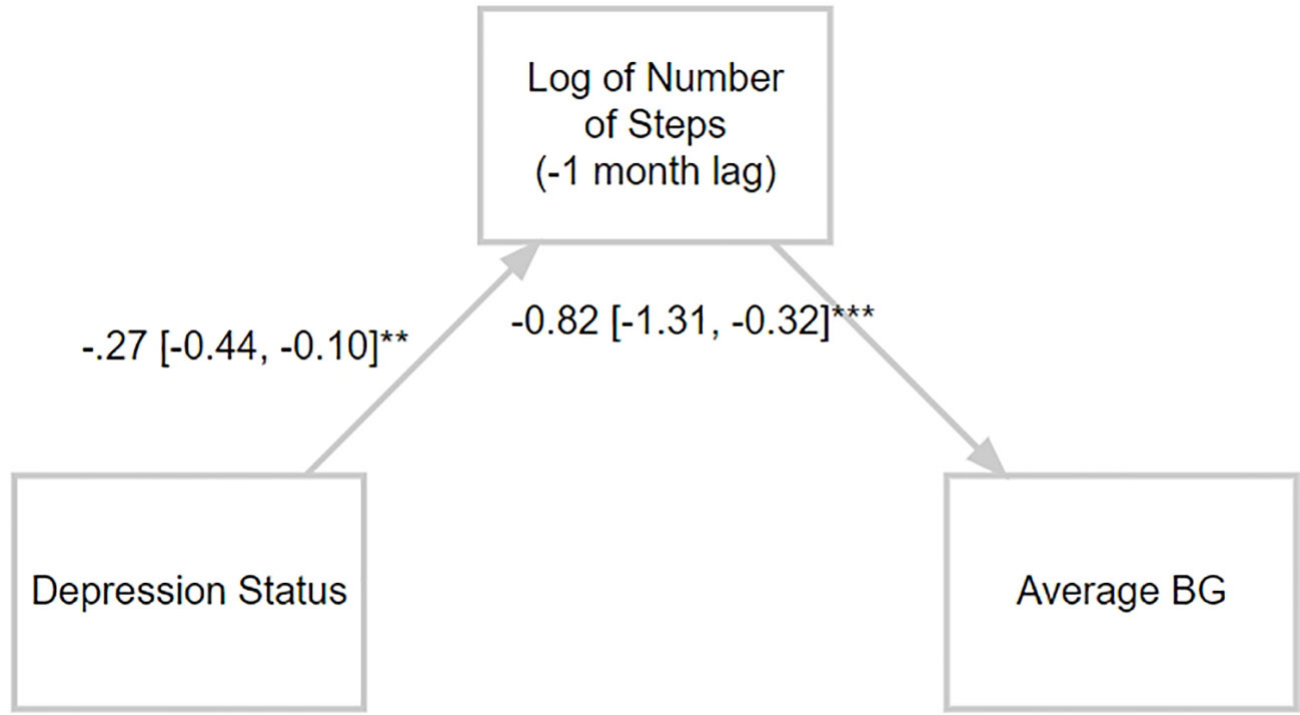
Mediation model. Self-reported depression is associated with the monthly average number of steps, which in turn predicts the following month’s average BG levels. The monthly average number of steps fully mediates the association between self-reported depression and monthly average BG (mg/dL).

Finally, the results of the mediation analysis demonstrated that there was a significant indirect effect of the depression status on the monthly average BG levels through the monthly average number of steps (M=.22, 95% CI [0.05, 0.45] P<.005) (**Figure 3**). The direct effect of depression status on the monthly average BG levels was insignificant (M=2.75, 95% CI [-2.2, 7.7], P=.29), indicating a full mediation.

Furthermore, a sensitivity analysis was conducted for the purpose of testing whether the mediation effect is stable over time. It was revealed that there was a significant indirect effect of the depression status on the monthly average BG levels through the monthly average number of steps at month 2 (M=.49, 95% CI [0.21, 0.82] P<.001); month 5 (M=.43, 95% CI [0.18, 0.74] P<.001); month 10 (M=.33, 95% CI [0.12, 0.61] P<.001) and month 12 (M=.29, 95% CI [0.09, 0.57] P<.005), stating that the mediation effect is stable over time.

### 3.4 The association of monthly average number of steps and monthly average blood glucose

**Figure 4:**
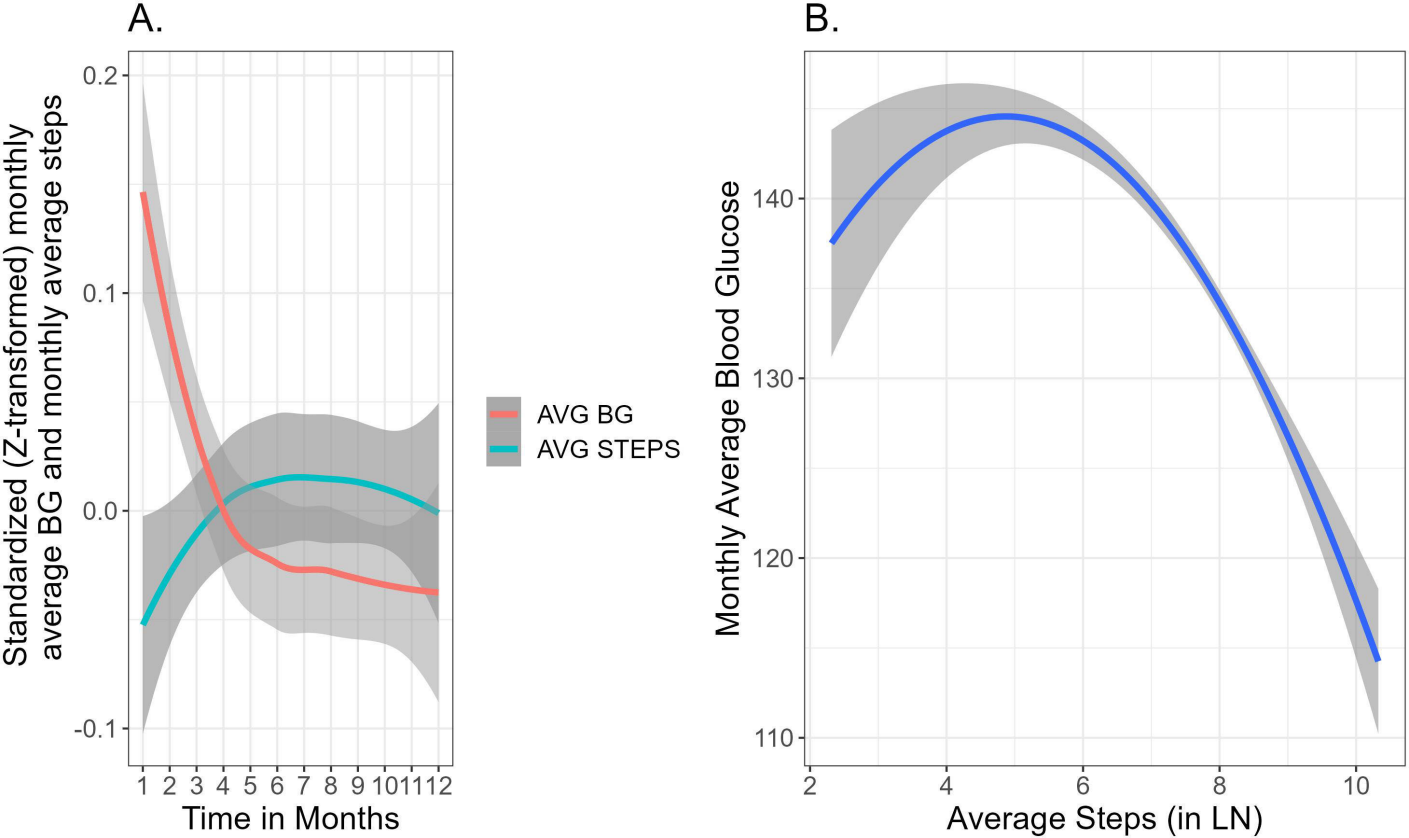
**(A)** Smoothed Standardized (Z-transformed) fluctuation of the monthly average BG and monthly average steps across 12 months of platform usage. Grey area represents 95% confidence intervals **(B)** Visualizes the association between the monthly average BG and monthly average steps a day (following a log transformation). The grey area around the lines represents 95% confidence intervals.

**Figure 5:**
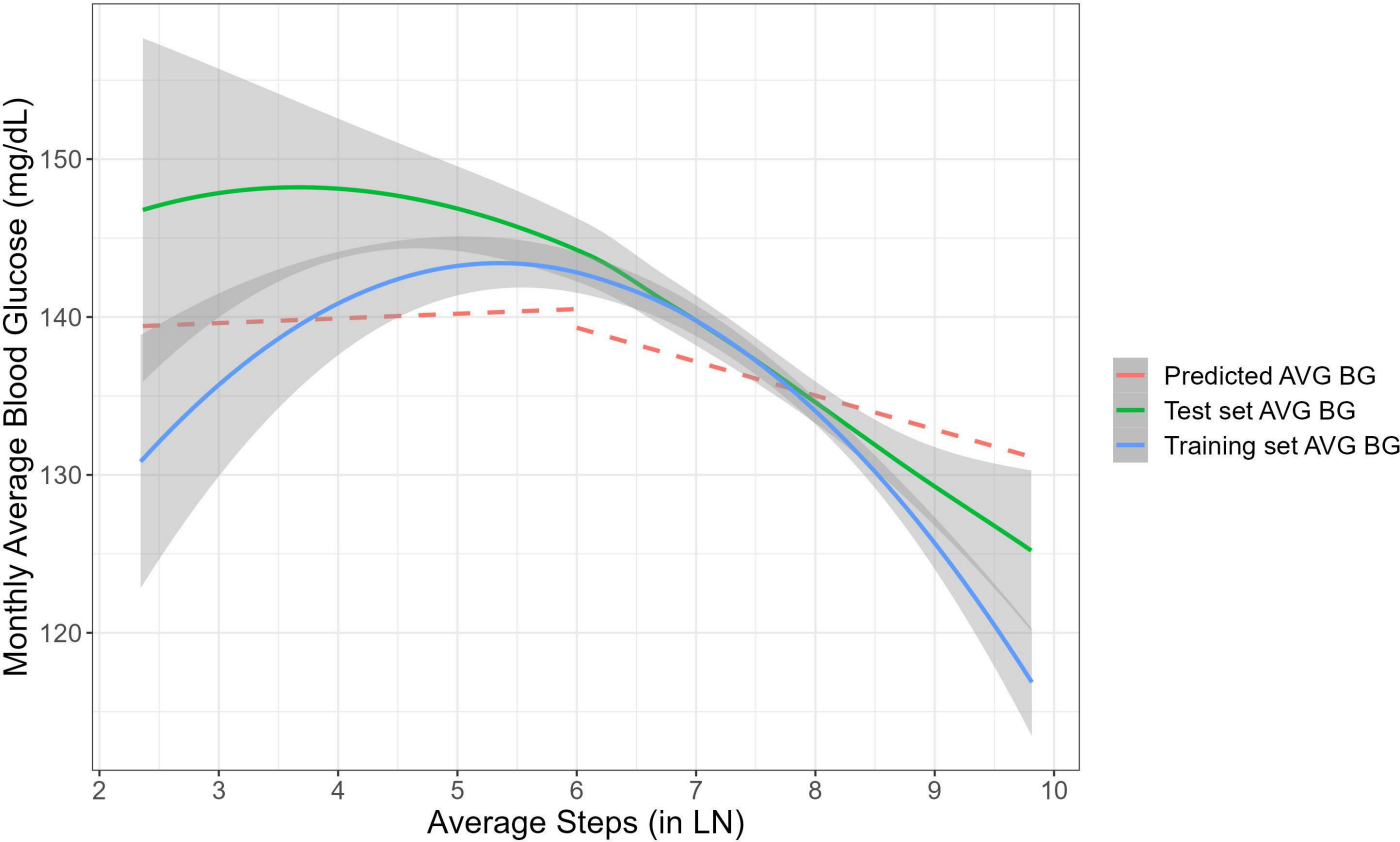
The non-linear association between the monthly average number of steps to the monthly average BG. Blue and green lines represent smoothing association between the variables for the training and test datasets. The grey area around each curve represents 95% confidence intervals. The red line shows the predictive piecewise mixed model applied on the test dataset.

**Figure 4A** demonstrates time-related fluctuations of the Z-transformed monthly average BG and monthly average number of steps, across 12 months of platform usage. **Figure 4B** visualizes the association between the monthly average BG with the monthly average number of steps. Piecewise mixed model analysis on the training data revealed a significant decrease in monthly average blood glucose for users with monthly average number of steps a day above 400 (B=-1.87, 95% CI [-3.17, -0.57], P<.01), while for users with monthly average number of steps a day less than 400 there were no significant changes in monthly average BG (B=.30, 95% CI [-0.24, 0.83], P=.28). **Figure 5** demonstrates the nonlinear association between the variables and predictive values of the piecewise mixed model applied on the training and test dataset. Training and test data have a similar temporal pattern, especially regarding the change-point in the slope and our model reflects it. The slight differences at the first slope (below 400 steps a day) can be explained by high variability due to the limited amount of data.

## 4 Discussion

Our analysis sheds light on the effect of self-reported depression status on BG levels over time. As shown in **Figure 2**, the monthly average BG levels were consistently higher in users with self-reported depression compared to those without. The ratio of people with depression in this study is 14% reflecting how the high prevalence of depressive disorders in people with diabetes, which ranges from 10% to 15% and is approximately twice as high as the prevalence of depression in people without diabetes (47–49). The piecewise-based model indicated that during the short-term adoption phase, while both users with depression and without depression show declines in average blood glucose levels, users who reported depression exhibited higher average BG levels compared to those without depression. In both cases we observed a significant decrease in BG levels during the initial 4 months of platform usage, followed by a stable period during months 4-12 of the monitoring. This finding underscores the importance of considering mental health factors in diabetes and prediabetes management. As with most chronic diseases, many factors contribute to the occurrence and treatment of diabetes, including depressive conditions (50).

Depression may contribute to poorer glycemic levels and necessitate tailored interventions to address both physical and psychological health needs (51–53). People living with diabetes and depressive disorders are at increased risk for earlier all-cause mortality compared to people living with diabetes without a history of depression (54, 55). Digital well-being interventions have the potential to mitigate prevalent barriers to psychological support in both non-clinical and clinical populations, including cost, stigma, and accessibility challenges (56, 57).

While depression can worsen diabetes outcomes by hindering self-care behaviors and increasing the risk of complications, having T2D might also contribute to the development of depression. Individuals with either type 1 or type 2 diabetes have a higher risk of developing depression and depressed people have a greater chance of developing type 2 diabetes (49, 58). Furthermore, the study found that the monthly average number of steps significantly mediated the effect of self-reported depression status on the following month’s average BG levels. The mediation effect reveals compelling insights into the complex interplay between depression, physical activity (measured by monthly average number of steps), and BG levels among individuals with diabetes or prediabetes and depression (**Figure 3**).

While there is a potential bidirectional relationship between depression and type 2 diabetes, the steps were not analyzed for their mediating effect on BG levels influencing depression levels in the following month. This analysis provides valuable insights into the mechanisms through which depression influences BG levels, highlighting the role of physical activity as a potential mediator. By promoting physical activity, interventions targeting depression may indirectly impact BG levels and improve diabetes management outcomes. Including both diabetes and prediabetes populations in a study analyzing the mediating role of walking activity in depression impacting blood glucose levels offers a beneficial factor. Prediabetes represents a stage where individuals have higher than normal blood glucose levels but not high enough to be classified as diabetes. Studying both prediabetes and diabetes allows us to examine the continuum of glucose metabolism. This holistic approach can provide insights into how early interventions (like physical activity) may impact blood glucose levels across different stages, potentially influencing preventive strategies for diabetes (59, 60). Moreover, individuals with prediabetes often share similar risk factors with those diagnosed with diabetes. Studying both populations together provides better understanding of common pathways linking depression, physical activity (such as walking), and blood glucose levels. This knowledge can aid in risk stratification and personalized interventions to prevent diabetes progression (61, 62). Individuals with diabetes and prediabetes frequently experience comorbidities such as depression, which can significantly impact their overall health outcomes. Analyzing the mediating role of walking activity in depression across both populations can inform integrated health management strategies that address mental health, physical activity, and glucose control simultaneously.

Firstly, the study identified a significant association between self-reported depression and the monthly average number of steps. This highlights the impact of depression on physical activity levels, suggesting that individuals with depression may engage in fewer steps compared to those without depression. This finding aligns with existing literature linking depression to decreased physical activity and highlights the importance of addressing mental health issues in promoting an active lifestyle among individuals with diabetes (63, 64).

Secondly, the analysis revealed a negative association between previous month physical activity and monthly average BG levels, conditioned on the effect of depression. This suggests that higher levels of physical activity are associated with lower BG levels underscoring the importance of regular exercise and walking activity as a key component of diabetes management that contributes to improved glycemic outcomes and overall health outcomes (65). Addressing the non-constant variance and the asymmetric error bars (**Figure 4**, **5**), we applied mixed-effects models which inherently indicate heteroscedasticity by modeling the variance structure at multiple levels simultaneously (66). They accommodate varying levels of variability across groups or conditions by estimating both fixed effects (mean structure) and random effects (variance structure) jointly. This approach provides valid estimates even when there are unequal variances across different groups or conditions (e.g. time points), ensuring robustness against heteroscedasticity in the data. Additionally, the non-constant variability between time points is also related to the fewer data points collected at certain time intervals, which can lead to increased variability in those estimates.

Indeed, experimental evidence has shown the effectiveness of mobile phone–based physical activity program on depression symptoms, through improvement of accessibility and encouragement of participation (67). Physical activity and exercise offer an effective alternative for treating depression by inducing interdependent changes in the brain, which foster a protective environment against depressive symptoms (68, 69). Moreover, the sensitivity analysis conducted to test the stability of the mediation effect over time revealed consistent findings. The moderated mediation model suggested that the mediation effect of physical activity on the relationship between self-reported depression and BG levels remains stable across various time points. This implies that the beneficial effects of physical activity on glycemic management persist over time. Physical activity is a key component of the therapeutic approach for people with diabetes (70). Regular involvement of individuals with diabetes in exercise programs could become a potential way to improve their quality of life reducing the economic expenditure for diabetes treatment and reducing complications that result from it (70, 71). Elevated physical activity and improved physical fitness have been shown to alleviate symptoms of depression and enhance health-related quality of life among individuals diagnosed with type 2 diabetes (72). This study highlights how management of physical activity with digital steps count targeting improved glycemia may work synergistically to enhance glycemic management and overall well-being and mental health in individuals with diabetes.

The presented results offer valuable insights into the dynamic relationship between physical activity, represented by monthly average number of steps, and improved glycemia, as reflected in the monthly average BG levels among users with diabetes. Previous research assumed this association to be linear, relying on limited sample size data (73) or real-life data with limited follow-up period (73, 74). The present study relied on a real-world larger sample size showing the negative association between monthly average BG levels and the monthly average number of steps exceeding 400 steps a day. This suggests a beneficial effect of higher levels of physical activity on blood sugar management, wherein increased step counts are associated with improved blood glucose levels. Conversely, no significant changes in the monthly average BG were observed among users with a monthly average number of less than 400 steps a day. This divergence in outcomes underscores the importance of achieving a certain threshold of physical activity to elicit favorable effects on blood glucose levels. The temporal patterns observed in both the training and test data, particularly regarding the change-point in the slope, indicate consistency and reliability in the model’s predictive capacity.

These findings hold significant clinical implications, emphasizing the pivotal role of physical activity in diabetes management. Healthcare providers should consider incorporating walking programs into routine diabetes care, particularly for patients with comorbid depression, to enhance both mental and physical health outcomes. Encouraging individuals to engage in performance and digital tracking of regular physical activity and, aiming to surpass the identified threshold of 400 steps a day may confer tangible benefits in terms of improved blood glucose levels for both depressed and non-depressed users. The non-linear effect that was tested revealed the minimum level of daily steps for a significant effect in blood glucose in population with average BMI of 32.5 (SD ±6.9) (**Figure 5**). Previous studies have published recommendations for daily steps accumulation that resulted in decreased blood glucose as well as systolic blood pressure in overweight population (75). Furthermore, the nonlinear nature of the association underscores the importance of personalized interventions tailored to individual activity levels and needs. Future research should investigate the impact of different speeds, durations, and rhythms of walking activity on blood glucose levels to enhance our understanding and application of mediation models and personalized behavioral interventions.

### 4.1 Limitations

As in all studies involving retrospective real-world data, groups were not randomly assigned, and treatment protocols were not prescribed. In addition, users who actively engaged with the Dario platform and consistently measured their blood glucose levels for a year likely represent a more motivated and engaged group compared to the general population of individuals with diabetes. This could limit the generalizability of the findings. Our analysis did not fully control for potential confounding factors such as socio-economic status and other lifestyle variables, which could have influenced both the likelihood of engaging with the Dario platform and the observed health outcomes. However, we included potential confounding factors collected on the platform in our model, such as age, gender, diabetes type, diabetes duration, insulin treatment, and weight. In this study, we relied on self-reported data regarding users’ comorbid conditions, specifically depression. While self-reporting is a common practical method in many observational and digital health studies, assessing depression solely through self-reporting without a clinically validated survey, has its limitations and may introduce misclassification bias. Nevertheless, studies have shown that self-reported diagnoses of depression are generally reliable (76–78). Though self-report measures should not replace in-person clinical evaluations, they can offer valuable supplemental information in a clinical setting and are likely to be increasingly utilized in the future (79). Moreover, the data collected in the study may not fully represent all patients with depression, and the timeliness of self-reporting can also vary. Nevertheless, for all users included in the analysis, we gathered self-reported medical data, including comorbid conditions such as depression, by having users select from a dropdown list of comorbidities.

In addition, there is a lack of information on whether users were taking antidepressant and antidiabetic medications. Future studies should incorporate clinically validated depression assessments and consider medication use to provide a more comprehensive understanding. There may be other physical activities besides walking that influence blood glucose levels and are not accounted for in the study. In addition, steps were synced weekly, daily activity levels were not fully captured in the analysis. We addressed this limitation by focusing on active users who consistently measured their blood glucose every week for a full year, their steps were consistently recorded on the platform and analyzed alongside blood glucose levels. Steps were synced to the platform weekly whenever the app was opened. It is certainly possible that people who chose to use pedometer and complete medical profile in the app were those who were the most motivated to change. Our inclusion criteria were designed to ensure that all participants in the analysis demonstrated commitment to managing their diabetes. This was evidenced by their consistent weekly blood glucose measurements throughout the 12-month study period, allowing for comprehensive analysis.

### 4.2 Conclusion

In conclusion, these findings emphasize the dynamic nature of BG management over time and highlight the importance of early intervention and ongoing support in diabetes management. Additionally, our findings underscore the importance of integrating physical activity, such as walking, into diabetes management protocols to improve both glycemic control and mental health in individuals with T2D and prediabetes. Further research is warranted to explore the neurophysiological mechanisms underlying the relationship between depression and glycemia and to develop targeted interventions aimed at improving health outcomes in this population. Future research could explore the effectiveness of integrated interventions targeting depression and physical activity in improving diabetes outcomes and long-term health trajectories.

## Data Availability

The datasets generated during and/or analyzed during this study are not publicly available due to company privacy policy but are available from the corresponding author on reasonable request according to the subject to company policies. Requests to access the datasets should be directed to yifat@dariohealth.com.
